# Exploring biomarkers for prognosis and neoadjuvant chemosensitivity in rectal cancer: Multi-omics and ctDNA sequencing collaboration

**DOI:** 10.3389/fimmu.2022.1013828

**Published:** 2022-12-09

**Authors:** Xiu-Feng Jiang, Bo-Miao Zhang, Fen-Qi Du, Jun-Nan Guo, Dan Wang, Yi-En Li, Shen-Hui Deng, Bin-Bin Cui, Yan-Long Liu

**Affiliations:** ^1^ Department of Colorectal Surgery, Harbin Medical University Cancer Hospital, Harbin, China; ^2^ Department of Neurology, The Second Affiliated Hospital of Qiqihar Medical University, Qiqihar, China; ^3^ Department of Anesthesiology, The Fourth Affiliated Hospital of Harbin Medical University, Harbin, China

**Keywords:** rectal cancer, cell-free DNA, genomic sequencing, multi-omics, prognosis, neoadjuvant chemotherapy

## Abstract

**Introduction:**

This study aimed to identified the key genes and sequencing metrics for predicting prognosis and efficacy of neoadjuvant chemotherapy (nCT) in rectal cancer (RC) based on genomic DNA sequencing in samples with different origin and multi-omics association database.

**Methods:**

We collected 16 RC patients and obtained DNA sequencing data from cancer tissues and plasma cell-free DNA before and after nCT. Various gene variations were analyzed, including single nucleotide variants (SNV), copy number variation (CNV), tumor mutation burden (TMB), copy number instability (CNI) and mutant-allele tumor heterogeneity (MATH). We also identified genes by which CNV level can differentiate the response to nCT. The Cancer Genome Atlas database and the Clinical Proteomic Tumor Analysis Consortium database were used to further evaluate the specific role of therapeutic relevant genes and screen out the key genes in multi-omics levels. After the intersection of the screened genes from differential expression analysis, survival analysis and principal components analysis dimensionality reduction cluster analysis, the key genes were finally identified.

**Results:**

The genes CNV level of principal component genes in baseline blood and cancer tissues could significantly distinguish the two groups of patients. The CNV of HSP90AA1, EGFR, SRC, MTOR, etc. were relatively gained in the better group compared with the poor group in baseline blood. The CNI and TMB was significantly different between the two groups. The increased expression of HSP90AA1, EGFR, and SRC was associated with increased sensitivity to multiple chemotherapeutic drugs. The nCT predictive score obtained by therapeutic relevant genes could be a potential prognostic indicator, and the combination with TMB could further refine prognostic prediction for patients. After a series of analysis in multi-omics association database, EGFR and HSP90AA1 with significant differences in multiple aspects were identified as the key predictive genes related to prognosis and the sensitivity of nCT.

**Discussion:**

This work revealed that effective combined application and analysis in multi-omics data are critical to search for predictive biomarkers. The key genes EGFR and HSP90AA1 could serve as an effective biomarker to predict prognose and neoadjuvant chemosensitivity.

## Introduction

The high incidence and poor prognosis of colorectal cancer (CRC) significantly impact the quality of life and economy of patients worldwide. It has also become the third leading cause of cancer death. Rectal cancer (RC) accounts for approximately one-third of all CRC cases (1). Multimodality therapies have been widely used in the clinical treatment of locally advanced patients due to advancements in pathology, imaging, genome sequencing technology, surgical technology, and instruments. Significant progress has been made in the last 30 years (2). Preoperative neoadjuvant radiotherapy and chemotherapy, total mesorectal excision, and postoperative adjuvant chemotherapy are the primary treatment methods that significantly reduce the local recurrence rate and improve the prognosis of patients. However, that strategy comes at the cost of quality of life (3). Despite this, only about 20% of RC patients treated with neoadjuvant chemotherapy (nCT) will have a complete response (CR) or partial response (PR) during preoperative evaluation or surgery (4). Most of the other patients benefited little from nCT after experiencing a series of toxic side effects. So far, the most significant clinical diagnosis and treatment challenge has been determining the best treatment strategy for each patient, enabling the individualized treatment, reducing side effects, and optimizing the quality of life. In addition, no biomarkers have been identified that can accurately predict the benefits of preoperative neoadjuvant therapy.

Recently, the rapid development of high-throughput sequencing technology has enabled next-generation sequencing (NGS) to detect various gene variants in cancer tissues based on DNA and RNA (5). In clinical applications, NGS-based DNA testing has demonstrated significant advantages in identifying clinically treatable genetic variants to guide patient treatment and prognostic-relevant adverse genomic variants before treatment (6). It also has clinical implications for predicting the outcome of chemotherapy. “Liquid Biopsy” has recently emerged as an accessible, convenient, and reproducible technique for real-time monitoring tumor patients by searching for circulating molecular markers in peripheral blood (7). In different types of cancer, biomarkers such as circulating tumor cells, microRNAs, and DNA have been investigated as potential diagnostic and prognostic markers for personalized therapies (8). This non-invasive blood-based test combined with NGS has significant advantages in patients who require preoperative nCT but cannot access fresh tumor tissue before surgery (9). It can investigate the changes and specific characteristics of the genome in patients at both the baseline and post-treatment level of neoadjuvant therapy, including single nucleotide variants (SNV), copy number variation (CNV) and tumor mutation burden (TMB).

Furthermore, The Cancer Genome Atlas (TCGA), a large genomic database, provides RNA-sequencing data from many RC patients before treatment. Numerous studies have demonstrated that genotyping based on RNA signatures in cancer tissues can accurately predict patient prognosis, chemosensitivity, and immunotherapy sensitivity, with high accuracy across multiple independent cohorts (10, 11). However, TCGA does not provide RNA-sequencing data of RC after treatment, and the genomes of untreated and treated tumors may differ significantly, limiting the analysis and prediction accuracy of neoadjuvant chemosensitivity for RC at the RNA level. A strong association between these two assays that target different genomic levels, such as the CNV level, will affect the RNA expression (12, 13). Meanwhile, increasing the copy number of a gene could be a mechanism for increasing protein expression (14). Therefore, effective combined application and analysis of them in multi-omics data are critical in the future search for predictive biomarkers for neoadjuvant chemosensitivity and to develop multimodality and individualized precision treatment.

In this study, we collected cancer tissues, adjacent tissues, and peripheral blood samples from 16 RC patients before and after nCT. Cell-free DNA (cfDNA) was isolated from the peripheral blood of patients, and the targeted gene capture panel sequencing was performed. The various gene variations in patients were examined, including SNV, CNV, TMB, copy number instability (CNI), and mutant-allele tumor heterogeneity (MATH). We also identified genes from the blood and tissue by which CNV levels can differentiate the response to nCT. The TCGA database was used to further evaluate the specific role of these genes and screen out the genes with significant differences in CNV in normal and cancer tissues. Then, we identified the genes whose expression levels were significantly correlated with CNV levels. The Clinical Proteomic Tumor Analysis Consortium (CPTAC) database was used for screening after the intersection of the screened genes from the above processes, and the key genes for predicting the prognosis and efficacy of nCT were finally obtained.

## Methods

### Sample collection

All patients received preoperative nCT, with capecitabine (950–1000 mg/m^2^) administered twice daily by oral gavage for 14 days and oxaliplatin (130 mg/m^2^) administered intravenously on the first day. Peripheral blood samples before and after nCT and surgically resected tumor tissue were collected from all patients. Following a quality control assessment, 16 patient samples met the criteria and were subjected to further analysis. There were seven patients in the CR group, one in the PR group, seven in the stable disease (SD) group, and one in the progressive disease (PD) group. The study was approved by the Ethics Committee of the Harbin Cancer Hospital Medical University, and all patients signed an informed consent document.

### CfDNA extraction and sequencing

Centrifugation at 1600 × g for 10 min separated the peripheral blood lymphocytes and plasma. The supernatant plasma was then transferred to a new 2 mL centrifuge tube and centrifuged at 16,000 × g for 10 min. MagMAXTM cfDNA isolation kit (Life Technologies, California, USA) was used to extract cfDNA in the plasma. Tiangen whole blood DNA kit (Tiangen, Beijing, PRC) was used to extract DNA from peripheral blood lymphocytes according to the manufacturer’s instructions. The DNA concentration was determined using either the Qubit dsDNA HS Assay kit or the Qubit dsDNA BR Assay kit (Life Technologies, California, USA).

Genomic DNA was sheared into 150–200 bp fragments with Covaris M220 Focused-ultrasonicatorTM Instrument (Covaris, Massachusetts, USA). Fragmented DNA and cfDNA libraries were constructed by KAPA HTP Library Preparation Kit (Illumina platforms) (KAPA Biosystems, Massachusetts, USA) as per the manufacturer’s instruction. A designed Genescope panel of 1086 genes (Genecast, Beijing, China) was used to capture the DNA libraries that included significant tumor-related genes. The captured samples were paired on an end sequencing Illumina HiSeq X-Ten.

### Basic analysis

Following quality control, clean data were aligned to the hg19 human genome using BWA 0.7.17 (15), and duplications were masked by Picard (v2.23.0) (16). Variants were called using VarScan (v2.4.2) (17) and annotated by ANNOVAR software (18). Somatic mutations were eliminated using the following steps: (i) Exclude the mutations annotated as synonymous SNVs or located in intergenic or intronic regions; (ii) Exclude mutations annotated with allele frequency ≥ 0.002 in the Exome Aggregation Consortium (ExAC) database (19) and the Genome Aggregation Database (20); (iii) Exclude mutations with strand bias, support reads < 5, and allele frequency < 0.05 in the tumor sample and allele frequency < 0.01 in the plasma sample. CNVs were called *via* cnvkit (v0.9.2) software using paired mode (21).

We used the principal components analysis (PCA) algorithm to perform dimensionality reduction and cluster analysis based on treatment efficacy information to screen for the potential genes that can predict nCT efficacy. The R package Complex heatmap (22) was performed to draw the landscape of genomic alterations in samples from different sources (patient’s baseline blood, post-chemotherapy blood, and CR tissue). The chromosomal locations of the genes identified by the above analyses were visualized by the R package “RCircos” (23). Furthermore, we also analyzed the differences in CNI, MATH, and TMB in baseline blood. CNI, TMB, and MATH differences were compared using the Mann–Whitney U test.

### CNI score calculation

The CNI score is a general measure of chromosomal instability (CIN) and is directly related to the regional chromosomal DNA ploidy (24, 25). To assess the extent of CIN, we quantified it using the CNI score. After GC content correction and normalization of target region length, read counts were transformed into log2 ratios (26). The log2 ratios were then converted into Z-score using Gaussian transformations versus a baseline group. The regions with Z-scores greater than the 95^th^ percentile plus twice the absolute standard deviation were defined as unstable regions. The CNI score was calculated by adding the Z-scores of unstable regions (25).

### MATH calculation

Somatic mutations with variant allele frequency (VAF) between 2% to 100% were included for MATH analysis. MATH was calculated by the following formula: Median absolute deviation of included somatic mutations/median VAF (27).

### TMB calculation

Absolute mutation counts were defined as the number of somatic mutations, and TMB was calculated with the formula: Absolute mutation counts * 1000000/Panel exonic base number (28).

### Preprocessing and analysisof TCGA RC samples

We included 165 RC patients in the TCGA database (Data Release 34.0, Release Date: July 27, 2022, https://tcga-data.nci.nih.gov/tcga/). The downloaded information included RNA expression data and CNV information. To search for genes with different CNV levels in normal and cancer tissues, we utilized a chi-square test to evaluate statistical significance.

To evaluate the specific role of therapeutic relevant genes identified by the RC blood samples sequencing results. We performed PCA analysis to extract the main components of those therapeutic relevant genes and then constructed a gene signature in TCGA cohort. Both principal components 1 and 2 were selected as signature scores. A method similar to the gene expression rank index was performed to define the nCT predictive score (nCTPS) of each patient: nCTPS = ∑PCA1i+∑PCA2i (i is the expression of therapeutic relevant genes). To identify the relative enrichment degree in biological processes of different groups, R packages “GSVA” (29) was used to perform enrichment analysis.

### Correlation analysis of genes at CNV and transcriptional levels

To investigate genes associated with CNV and transcript levels, we extracted the RNA expression matrix of the above genes with significantly different CNVs. We divided all samples into four groups: single deletion, normal, single gain, and amplification groups. The R software (version 4.0.5) was then used for statistical analysis. Differences between the two groups were compared using the Wilcoxon test, while comparisons between more than two groups were performed using the Kruskal-Wallis test. In all results, p < 0.05 was considered statistically significant.

### Identification of potential genes for predicting efficacy of neoadjuvant chemotherapy

After obtaining the genes whose transcriptional levels were associated with CNVs, we intersected these genes with the genes obtained from PCA and cluster analysis that might predict the efficacy of nCT. These genes in the intersection have great potential and value for predicting the efficacy of nCT. Then, to verify the effect of these intersecting genes on chemosensitivity, we performed a drug sensitivity prediction analysis. Gene expression data and chemotherapeutic drug response data were downloaded from CellMiner™ (https://discover.nci.nih.gov/cellminer/), these data were from the same batch. We deleted drugs that without FDA-approval or clinical trials, and selected chemotherapy drugs for RC. Then we extracted the genes expression data, and analyzed the correlation between their expression and drug sensitivity.

To further screen and validate the key genes at the protein level, we downloaded the proteomic cohort of RC from the CPTAC database. Meanwhile, a web tool, the University of ALabama at Birmingham CANcer data analysis Portal (UALCAN) (http://ualcan.path.uab.edu/), was chosen to be used, which integrates the proteomic data of all tumor samples from the CPTAC database. We downloaded the immunohistochemical staining images from The Human Protein Atlas project (https://www.proteinatlas.org/). Each sample is represented by 1 mm tissue cores (30). In colon cancer (CC) and RC samples, we analyzed the differential expression of the screened genes in normal and cancer issues, their impact on prognosis, and the correlation between their transcriptional levels and protein levels, respectively. To distinguish the high and low expression groups associated to prognosis, the best cut-off value was estimated by R package “maxstat” (31). Genes with significantly different results in multiple aspects were finally identified as the key genes. Finally, DisNor database (32) (https://disnor.uniroma2.it/) was used to analyze the up- and downstream binding sites and causal interaction of the key genes.

## Results

### Cluster analysis of SNV and CNV in three types of samples

**Figure 1:**
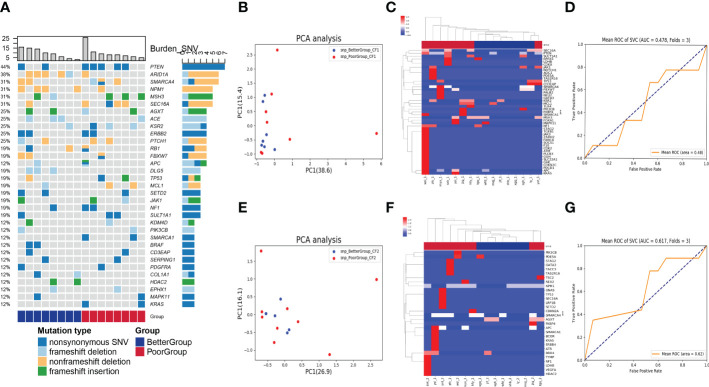
Analysis of SNV in blood samples before and after nCT on therapeutic response. **(A)** The heatmap showed genes with SNV detected in the baseline blood according to the frequency of occurrence from high to low. The columns represent the RC patients and the rows represent the genes. The colors on the right represent the efficacy groups and mutation type. **(B)** The PCA dimensionality reduction and cluster analysis for SNP level in baseline blood. **(C)** The heatmap demonstrated the SNP level of the principal component genes in baseline blood. **(D)** The receiver operating characteristic curve for distinguishing the efficacy groups in baseline blood (AUC = 0.48). **(E)** The PCA dimensionality reduction and cluster analysis for SNP level in the blood after nCT. **(F)** The heatmap demonstrated the SNP level of the principal component genes in the blood after nCT. **(G)** The receiver operating characteristic curve for distinguishing the efficacy groups in blood after nCT (AUC = 0.62).

First, we used the heatmap to present the genes with SNV detected in the baseline blood in order of frequency of occurrence from high to low, which were PTEN, ARID1A, SMARCA4, NPM1, MSH3, SEC16A, AGXT, ACE, KSR2, ERBB2, PTCH1, RB1, FBXW7, APC, DLG5, TP53, MCL1, SETD2, JAK1, NF1, SULT1A1, KDM4D, PIK3CB, SMARCA1, BRAF, CD3EAP, SERPING1, PDGFRA, COL1A1, HDAC2, EPHX1, MAPK11, and KRAS. The nonsynonymous SNV was the most common mutation type among the genes in the heatmap, occurring almost in every gene, followed by frameshift deletion, non-frameshift deletion, and frameshift insertion (**Figure 1A**). We found that the SNP level of the principal component genes in baseline blood was less effective for distinguishing the two groups by PCA dimensionality reduction cluster analysis (**Figures 1B, C**), with an area under the receiver operating characteristic curve (AUC) of only 0.48 (**Figure 1D**). In addition, after chemotherapy, the SNP level of the principal component genes in blood was less effective in distinguishing the two groups (**Figures 1E, F**), with an AUC of only 0.62 (**Figure 1G**).

**Figure 2:**
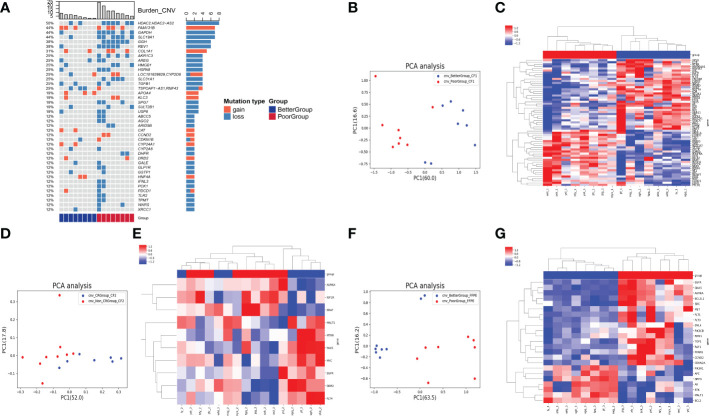
Analysis of CNV in 3 types of samples on therapeutic response. **(A)** The heatmap showed genes with CNV in descending order of occurrence frequency. The columns represent the RC patients and the rows represent the genes. The colors on the right represent the efficacy groups and CNV type. **(B)** The PCA dimensionality reduction and cluster analysis for CNV level in baseline blood. **(C)** The heatmap demonstrated the CNV level of the principal component genes in baseline blood could significantly distinguish the two groups of patients. **(D)** The PCA dimensionality reduction and cluster analysis for CNV level in the blood after nCT. **(E)** The heatmap demonstrated the CNV level of the principal component genes in the blood after nCT. **(F)** The PCA dimensionality reduction and cluster analysis for CNV level in cancer tissues obtained from surgery. **(G)** The heatmap demonstrated the CNV level of the principal component genes in cancer tissues could significantly distinguish the two groups of patients.

In the CNV analysis, the genes with CNV in descending order of occurrence frequency are: HDAC2, FAM131B, GAPDH, SLC19A1, GGH, REV1, COL1A1, AKR1C3, AREG, HMGB1, HSPA8, CYP2D6, SLC31A1, TGFB1, RNF43, APOA4, KLC3, SPG7, SULT2B1, USP6, ABCC5, AGO2, ARID5B, CAT, CCND2, CDKN1B, CYP24A1, CYP2A6, DHFR, DRD2, GALE, GLP1R, GSTP1, HNF4A, IFNL3, PCK1, PDCD1, TLR2, TPMT, WARS, XRCC1 (**Figure 2A**). Most of the top 40 genes with CNV occurrence have copy number deletion (32/41), with a small number of genes having copy number gain (13/41). Meanwhile, in the poor group, most patients’ genes with CNVs exhibited copy number deletion (**Figure 2A**). We identified that the CNV level of principal component genes in baseline blood could significantly distinguish the two groups of patients using PCA dimensionality reduction cluster analysis (**Figure 2B**). The CNVs of DPYP, IL7R, MTRR, HSP90AA1, MAP3K7, VHL, EGFR, ETV4, FLT4, CREBBP, CHEK2, NSD1, PPARG, DDR2, MUTYH, KDR, NCOA1, GNAQ, PTGS2, ETV1, PAX5, AR, BTK, XPC, MPL, GNA11, SRC, FGGR3, NOTCH1, FANCA, JAK3, ITGB2, mTOR, and ARNT were relatively gained in the better group compared with the poor group. In contrast, the CNVs of ABL1, ARID1A, CCND2, TSC1, SYK, ERBB3, SMO, NOTCH2, SETD2, IGF1R, AURKA, PDGFRA, IDH1, PALB2, BRAF, PIK3CB, ERCC4, NRAS, MDM4, NF1, APC, FBXW7, ESR1, MITF, CCND1, CDH1, and PTCH1 were relatively gained in the poor group compared with the better group (**Figure 2C**).

However, after clustering by PCA dimensionality reduction in the blood samples after nCT (**Figure 2D**), the CNV level of the principal component genes could not significantly differentiate the two groups (**Figure 2E**). The PCA analysis of cancer tissues obtained from surgery (**Figure 2F**) revealed that the CNV levels of EGFR, GNAS, AURKA, BCL2L1, SRC, MET, FLT1, FLT3, EML4, PIK3CB, RRM1, TOP1, RAF1, PPARG, CCND2, CDKN2A, PIK3R1, APC, DPYD, AR, BTK, MALT1, and BCL2 could significantly differentiate the two groups of patients (**Figure 2G**).

### Analysis of chemotherapy-related loci, CNI, MATH, and TMB in baseline blood

**Figure 3:**
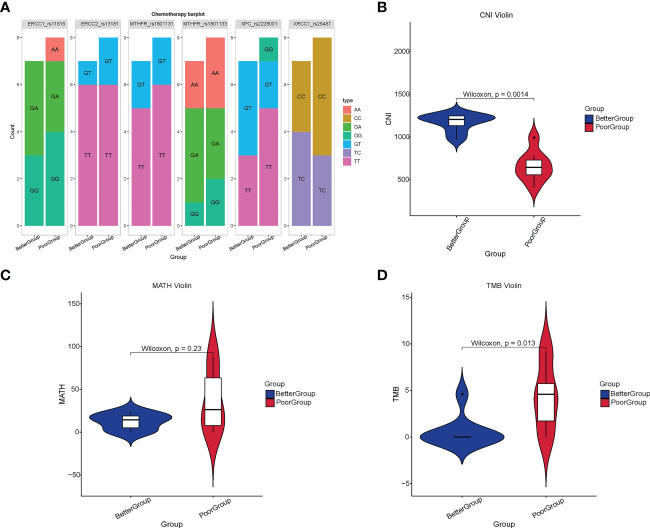
Analysis of chemotherapy-related loci, CNI, MATH, and TMB in different therapeutic response groups. **(A)** The commonly used target genotypes of chemotherapy in 16 RC patients. **(B)** The CNI value in the better group was significantly higher than that in the poor group (*p* = 0.0014) **(C)** There was no significant difference in the MATH value between the two groups (*p* = 0.23). **(D)** The TMB in the poor group was significantly higher than that in the better group (*p* = 0.013).

In addition to baseline blood samples, we examined other relevant indicators. The findings revealed that the commonly used chemotherapy target genotypes, including RCC1, ERCC2, MTHFR, XPC, and XRCC1, were not significantly different between the two groups of patients (all *p* > 0.05) (**Figure 3A**). CNI measures genomic instability related to regional chromosomal DNA ploidy (24). The calculation of the CNI value by bioinformatics analysis demonstrated that these values were significantly higher in the better group than in the poor group (*p* = 0.0014) (**Figure 3B**). The MATH value is a scoring method used to estimate tumor heterogeneity, and its higher value indicates more heterogeneity (33). MATH value calculation illustrates no significant difference between the two groups (*p* = 0.23) (**Figure 3C**). However, in the case of TMB, it was identified that TMB was significantly higher in the poor group than in the better group (*p* = 0.013) (**Figure 3D**).

### Evaluation of the specific role of therapeutic relevant genes in the TCGA cohort

Given the purpose of our study was to identify the biomarkers and predict therapeutic response before nCT and surgery, we selected the results from the samples available before treatment, that is, the therapeutic relevant genes identified in the baseline blood for subsequent analysis.

**Figure 4:**
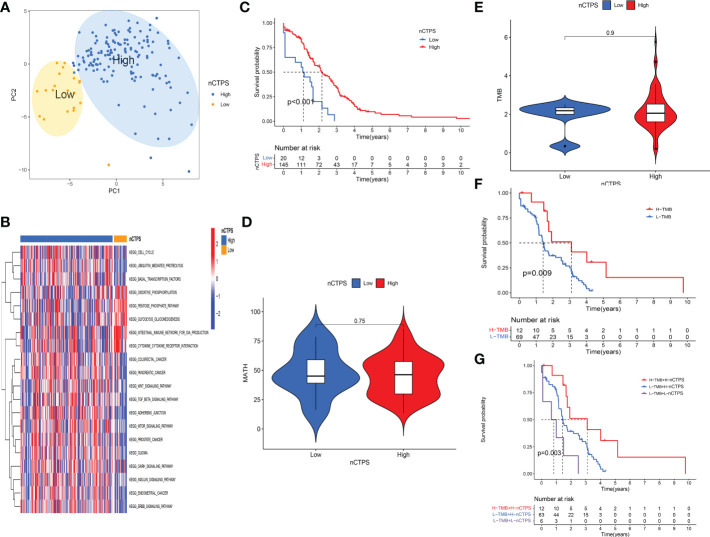
Evaluation of the specific role of therapeutic relevant genes in the TCGA cohort. **(A)** Kaplan-Meier curves showed the overall survival difference between high and low nTCPS groups (*p* < 0.001). **(B)** The GSVA analysis between high and low nTCPS groups. Red and blue represented relative enrichment degree of activated and inhibited pathways, respectively. **(C, D)** There were no significant differences in TMB (*p* = 0.9) **(C)** and MATH (*p* = 0.75) **(D)** between high and low nTCPS groups. **(E)** PCA scatter plot showed that two groups of patients can be separated by the PCA clustering analysis. **(F)** Kaplan-Meier curves showed the overall survival difference between high and low TMB groups (*p* = 0.009). **(G)** The stratified prognostic analysis showed that the synergistic effect of the TMB and nTCPS on the prognosis (*p* = 0.003).

To evaluate the specific role of these genes, we used PCA to constructed a gene signature in TCGA cohort, and defined the results as nCTPS. Next, we evaluated the value of the nCTPS in predicting prognosis. After obtaining the best cut-off value through R package “maxstat” (31), we distributed the patients from cohort into high and low nCTPS groups. We used PCA scatterplot to show the distribution of two groups of patients. It can be seen from the figure that the two groups of patients can be also separated by the PCA clustering (**Figure 4A**). We performed Gene Set Variation Analysis (GSVA) between the two groups. The results showed that enrichment of pathways varied significantly between the two groups, including ErbB signaling pathway, GnRH signaling pathway, mTOR signaling pathway, Wnt signaling pathway, etc. (all q-values < 0.05) (**Figure 4B**). There was a significant difference in prognosis between the two groups (*p* < 0.001) (**Figure 4C**). Furthermore, we calculated the MATH and TMB values in TCGA rectal cancer patients. We found that there was no significant difference in MATH and TMB between the high and low score groups (**Figures 4D, E**). There was a significant difference in the prognosis of patients in the high and low TMB groups (p = 0.009) (**Figure 4F**). Taking the synergistic effect of the TMB and nCTPS on the prognosis, we performed a stratified prognostic analysis. The results indicated that the nCTPS could be a potential prognostic indicator, and the combination with TMB could further refine prognostic prediction for patients (p = 0.003) (**Figure 4G**).

### Transcript level combined with CNV to further identify genes associated with chemosensitivity

**Figure 5:**
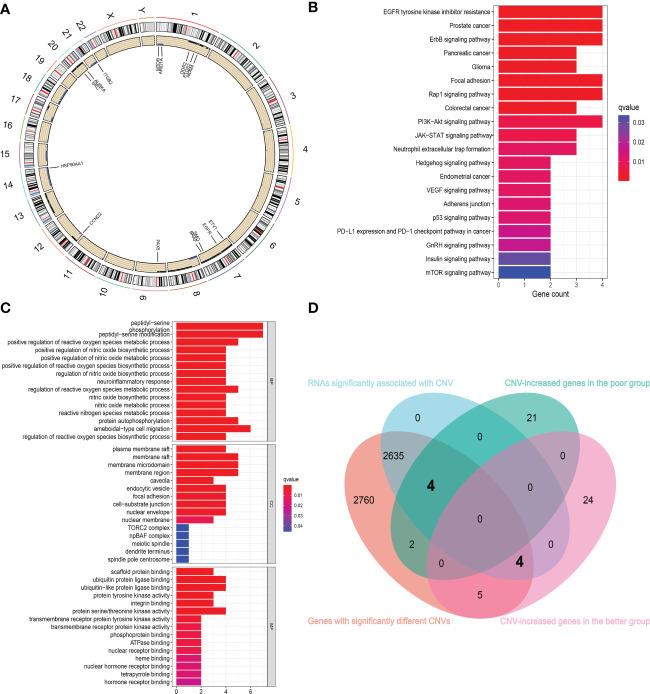
Combining with TCGA cohort to further identify the genes associated with chemosensitivity. **(A)** The chromosomal locations of 15 overlapped genes and CNV alterations. **(B, C)** The KEGG **(B)** and GO **(C)** enrichment analysis of 15 overlapped genes showed that they are significantly enriched in many energy metabolism pathways and classic cancer-related signaling pathways. **(D)** The Venn diagram showed the gene sets screened from different analyses.

We first identified 5410 genes in the RC data from TCGA, whose CNVs differ significantly between cancer and normal tissues (all adjust *p* < 0.05). Fifteen of them overlapped with the genes that can differentiate sensitivity to nCT identified using the RC baseline blood sample sequencing results. **Figure 5A** depicts the chromosomal locations of these genes and CNV alterations, with most of these genes located in Chromosomes 1 and 7. The Gene Ontology (GO) and Kyoto Encyclopedia of Genes and Genomes (KEGG) enrichment analysis of these genes demonstrated that they were significantly enriched in many energy metabolism pathways and classic cancer-related signaling pathways, including regulation of reactive oxygen species biosynthetic process, protein autophosphorylation, EGFR tyrosine kinase inhibitor resistance, ErbB signaling pathway, P53 signaling pathway, and mTOR signaling pathway (**Figures 5B, C**). The overlapped enriched pathways among GSVA analysis, GO and KEGG enrichment analysis include ErbB signaling pathway, GnRH signaling pathway, mTOR signaling pathway, pancreatic cancer, colorectal cancer, endometrial cancer, adherens junction signaling pathway, etc. These overlapped signaling pathways are closely related to both tumor progression and drug resistance (34, 35). Therefore, we performed a chemosensitivity analysis on these genes. The findings revealed that the increased expression of genes with CNV levels relatively gained in the poor group was associated with decreased sensitivity to multiple chemotherapeutic drugs, including AURKA and BRAF (**Supplementary Figure 1A**). However, in the better group, the increased expression of genes with CNV relatively gained was associated with increased sensitivity to multiple chemotherapeutic drugs, including EGFR, SRC, and HSP90AA1 (**Supplementary Figure 1B**).

Subsequently, we extracted the expression of RNAs with significantly different CNVs in normal and cancer tissues, analyzed the data, and obtained 2643 genes with significant differences in expression levels at different CNV levels. Then, using the sequencing results of RC blood samples, we intersected these genes with the genes that can distinguish the sensitivity to nCT and obtained eight intersecting genes (**Figure 5D**). The CNV levels of HSP90AA1, EGFR, SRC, and mTOR were higher in the better group compared to the poor group. In contrast, the CNV levels of ARID1A, AURKA, BRAF, and MDM4 were higher in the poor group compared to the better group. The **Supplementary Figures 2A–H** represents the changes in RNA expression of these eight genes at different CNV levels (all *p* < 0.05).

### Transcript level combined with protein level to finalize the key genes

**Figure 6:**
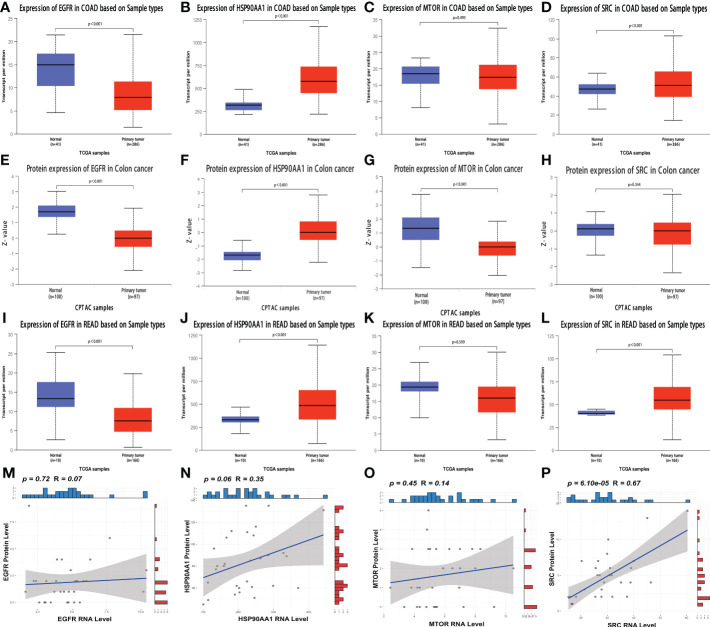
Analysis of the expression and correlation of key genes at the protein and transcript levels **(A–D)** The transcriptional expression of EGFR **(A)**, HSP90AA1 **(B)**, MTOR **(C)** and SRC **(D)** in normal and CC tissues. **(E–H)** The protein level of EGFR **(E)**, HSP90AA1 **(F)**, MTOR **(G)** and SRC **(H)** in normal and CC tissues. **(I–L)** The transcriptional expression of EGFR **(I)**, HSP90AA1 **(J)**, MTOR **(K)** and SRC **(L)** in normal and RC tissues. **(M–P)** The correlation analyses between RNA expression and protein level of EGFR **(M)**, HSP90AA1 **(N)**, MTOR **(O)** and SRC **(P)** in RC patients.

A publicly proteomic database was used to analyze expression differences, prognosis, and protein-transcript level correlations for identifying and validating key genes. Because of the scarcity of publicly available RC proteomic data and lack of data on normal tissues, colon cancer (CC) and RC are inseparable from gastrointestinal cancers, with close correlations in various aspects (36). Therefore, we included CC proteomic data in our analysis. At the transcriptional level of CC, EGFR (**Figure 6A**), HSP90AA1 (**Figure 6B**), and SRC (**Figure 6D**) expression levels were significantly different between normal and cancer tissues (all *p* < 0.05). However, there was no significant difference between normal and cancer tissues in MTOR (p = 0.493) (**Figure 6C**). At the protein level, the expression levels of EGFR (**Figure 6E**, **Supplementary Figures 3A–F**), HSP90AA1 (**Figure 6F**, **Supplementary Figures 3G–L**), and mTOR (**Figure 6G**) were significantly different between normal and cancer tissues (all *p* < 0.05). There was no significant difference between normal and cancer tissues in SRC (p = 0.344) (**Figure 6H**). The expression levels of EGFR (**Figure 6I**), HSP90AA1 (**Figure 6J**), and SRC (**Figure 6L**) at the transcriptional level of RC were significantly different between normal and cancer tissues (all *p* < 0.05). However, there was no significant difference between normal and cancer tissues in MTOR (p = 0.599) (**Figure 6K**). Furthermore, the RNA expression of SRC was positively correlated with the protein expression (r = 0.67, *p* < 0.001) (**Figure 6P**), and the RNA expression of HSP90AA1 was positively correlated with the protein expression, and the statistical significance approached significant (r = 0.35, *p* = 0.06) (**Figure 6N**). Nevertheless, there was no significant correlations between RNA and protein level in EGFR (**Figure 6M**) and MTOR (**Figure 6O**).

**Figure 7:**
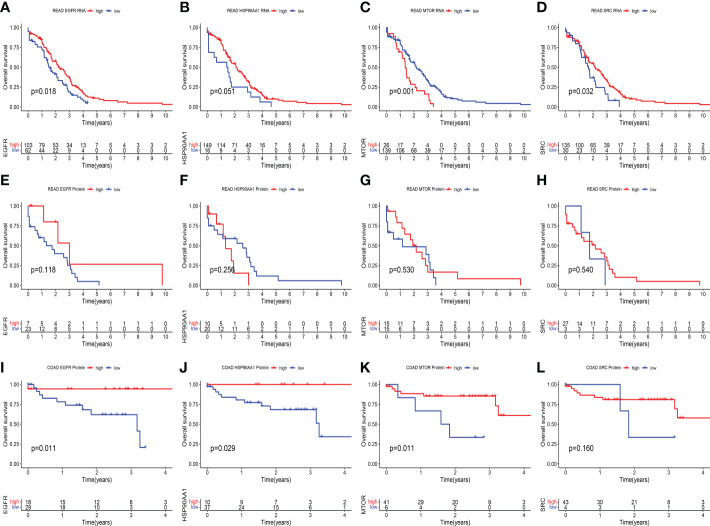
Kaplan–Meier survival analysis in different expression groups of key genes at the protein and transcript levels. **(A–D)** At transcriptional level of RC, Kaplan-Meier curves showed the overall survival difference between high and expression group of EGFR (*p* = 0.018) **(A)**, HSP90AA1 (*p* = 0.051) **(B)**, MTOR (*p* = 0.001) **(C)** and SRC (*p* = 0.032) **(D)**. **(E–H)** At protein level of RC, Kaplan-Meier curves showed the overall survival difference between high and low expression group of EGFR (*p* = 0.118) **(E)**, HSP90AA1 (*p* = 0.256) **(F)**, MTOR (*p* = 0.530) **(G)** and SRC (*p* = 0.540) **(H)**. **(I–L)** At protein level of CC, Kaplan-Meier curves showed the overall survival difference between high and expression group of EGFR (*p* = 0.011) **(I)**, HSP90AA1 (*p* = 0.029) **(J)**, MTOR (*p* = 0.011) **(K)** and SRC (*p* = 0.160) **(L)**.

In terms of prognosis, at the transcriptional level of RC, the high expression group of EGFR (**Figure 7A**) and SRC (**Figure 7D**) had a significantly better prognosis than the low expression group (all *p* < 0.05). The patients with high HSP90AA1 expression seem to have a better prognosis than those with low HSP90AA1 expression, this difference approached statistical significance (*p* = 0.051) (**Figure 7B**). In contrast to the above results, the high expression group of MTOR had a significantly poor prognosis than the low expression group (p = 0.001) (**Figure 7C**). However, there was no significant difference in prognosis between the high and low expression groups of these four genes at the protein level (all *p* > 0.05) (**Figures 7E–7H**). The lack of positive results could be attributed to the small sample size. The prognosis of the high expression group of EGFR (**Figure 7I**), HSP90AA1 (**Figure 7J**), and mTOR (**Figure 7K**) at the protein level of CC was significantly better than that of the low expression group (all *p* < 0.05). However, there were no significant difference in survival between high and low SRC expression group (p = 0.16) (**Figure 7L**).

**Figure 8:**
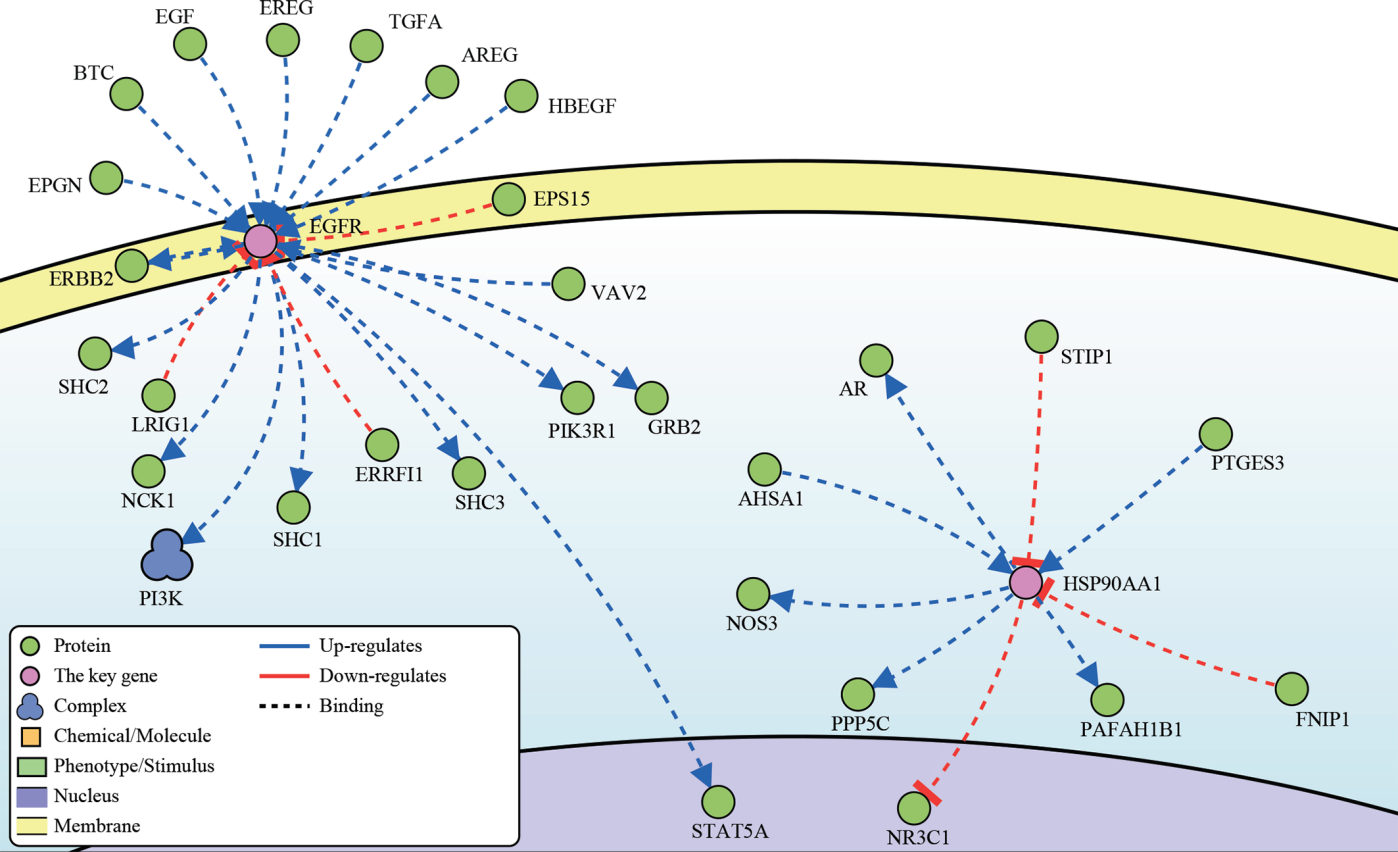
The up- and downstream binding sites and causal interaction of the key genes’ analysis in DisNor.

Altogether, the genes with significant differences in multi-omics and multiple aspects were EGFR and HSP90AA1. Therefore, they were identified as the key predictive genes related to prognosis and the sensitivity of nCT. DisNor database (32) revealed the EGFR and HSP90AA1 up and downstream binding sites and their causal interaction (**Figure 8**). The up-regulated binding sites of EGFR included PI3K, PI3K3R1, TGFA, SHC1, and SHC3. In contrast, LRIG1 and ERRFI1 were the EGFR down-regulated binding sites. AHSA1 and PTGES3 were the up-regulated, while STIP1 and FNIP1 were the down-regulated binding sites of HSP90AA1, respectively. These findings are critical for understanding the mechanisms and signaling pathways of the key genes involved in drug resistance and identifying potential therapeutic targets.

## Discussion

Researchers and clinicians extensively investigate locally advanced RC (LARC) because of its high morbidity and distant metastasis rate (37). Researchers have proposed a new treatment, nCT, to improve the prognosis of such patients. CAO/ARO/AIO-94, CAO/ARO/AIO-04, MRC CR07, and NCIC-CTG C016 all confirmed that nCT could significantly improve the surgical resection rate of LACR, decrease the local recurrence rate, and reduce the occurrence of adverse reactions (38–40). However, numerous studies have found significant differences in patients’ ability to respond to nCT. Approximately 50% of patients had a PR, 8–20% achieved pathological CR, and about 20% demonstrated resistance to nCT (41–45).

The response heterogeneity to nCT has resulted in overtreatment, increased cytotoxicity, or economic pressure and may even result in disease progression due to delayed radical surgery. Finding high-performance markers to distinguish which patients can benefit from nCT is thus an important means of promoting the standardization of LACR treatment. Recent studies have identified potential markers, such as DNA mutation, DNA methylation, circulating tumor cells, tumor immune microenvironment, and microRNA (46). However, these markers cannot be widely used due to sensitivity and specificity issues. Therefore, presently there is a need to identify new markers. We identified markers in blood samples that could predict patients’ response to nCT and proved that these markers could be used at DNA, transcription and protein levels. This multi-omics-validated marker has high reliability and stability, convenient sampling, and is not constrained by detection technology. They are highly reliable biomarkers that need further thorough investigations.

At the DNA level, we identified not only the genes associated with nCT response rates but also that CNV, CNI score, and TMB were potential markers, whereas SNP and MATH were not. These findings implied that CNV, CNI, and TMB can be used to predict the patient’s ability to respond to nCT (4, 47, 48). In this study, CNI score was used to assess the extent of CIN. Although CIN is ubiquitous in human cancers, its role in tumor evolution is complex and contradictory (49). On the one hand, CIN and complex aneuploidy are associated with resistance to anticancer drugs, such as paclitaxel, in tumor-derived cell lines and clinical settings (50, 51). Conversely, high CIN levels indicate enhanced sensitivity to cytotoxic therapies such as 5-fluorouracil and cisplatin in rectal (52), breast (53) and ovarian cancers (51). Induction of whole chromosome missegregation makes transplanted glioblastoma tumor sensitive to radiotherapy (54). In our analysis, the CNI value was significantly higher in the better group than in the poor group (*p* = 0.0014), which was consistent with previous research. This drastically different effect stems from the complex phenotypes conferred by CIN on cancer cells and the tumor microenvironment (49). CIN can serve as a genomic source of innate immune activation. For example, chromosome segregation errors can directly lead to the activation of immune signaling pathways (55). In addition, CIN can also act as a trigger of tumor immune editing. Chromosomal segregation errors caused by CIN at the early stages of tumorigenesis activate the cGAS-STING pathway, which functions as an innate cellular defense against viral infection (56).

We then perform further analysis at transcript and protein levels. We confirmed that EGFR and HSP90AA1 were the genes with significant differences in multi-omics and multiple aspects. SRC and mTOR did not have this relationship and only differed in a single omics. This demonstrated that EGFR and HSP90AA1 are multi-omics-validated markers that can predict patient response to nCT and require further investigation.

HSP90AA1 is a stress-inducible member of the HSP90 family. It can regulate the tumor-promoting process of many proto-oncogenes, including c-Myc, and is also associated with the malignant tumor phenotype, tumor growth, proliferation, invasion, and chemotherapy resistance (57–59). However, no studies have suggested that HSP90AA1 can be used as a prognostic or predictive marker for nCT. The present study is the first to describe the potential of this molecule as a predictive marker for nCT, which requires further research. EGFR is an important member of the erbB family that significantly regulate cell proliferation, differentiation, division, survival, and cancer development.

Therefore, EGFR is an important target for targeted therapy (60). Recent studies established that EGFR can be used as a prognostic and predictive molecular marker for nCT in various cancers (61–63). A study about RC demonstrated that circulating EGFR could be used as a potential biomarker for predicting pCR (61). These findings are consistent with our results, which indicated that EGFR could be used as an important target and a sensitive marker to guide RC treatment. SRC and SRC-family protein kinases are proto-oncogenes involved in cell morphology, motility, proliferation, and survival (64). SRC has recently been associated with the prognosis and recurrence of various tumors (65–67). Our findings demonstrated that SRC is not only associated with tumor prognosis but can be used to predict the patient’s ability to respond to nCT. mTOR is a serine/threonine kinase regulating various cellular metabolic processes such as protein synthesis and inactivation. mTOR can activate somatic mutations during tumorigenesis, making it an important therapeutic target (68). Some studies have illustrated that components of mTOR-related pathways can be used to predict tumor response to nCT (69, 70). Zhu et al. described in RC that GOLPH3 could predict patient sensitivity to nCT, which plays an important role in mTOR-related pathways (69). However, these studies have not directly demonstrated that mTOR could predict patient sensitivity to nCT. It is worth noting that the present study shows this for the first time.

To pave the way for future studies, we conducted a survival analysis. We described that the above markers could be used as prognostic markers for nCT in CRC, confirming the significance of these markers. We also investigated the signaling pathways of the above markers and found that they can function in multiple signaling pathways. EGFR was enriched in CRC, EGFR tyrosine kinase inhibitor resistance, the FoxO signaling pathway, the Oxytocin signaling pathway, the JAK-STAT signaling pathway, the Pl3K-Akt signaling pathway, and hedgehog signaling pathway. These pathways are of significant value in the occurrence and development of tumors, cancer stem cells, immune response, drug sensitivity, and drug resistance (71–75). Therefore, in-depth research on the markers mentioned above will help explain tumor pathogenesis further and improve patients’ prognosis.

However, several limitations remain as follows: Due to the small sample size of proteomic sequencing for RC in TCGA and some missing expression data of individual proteins, fewer analysis can be done and reliable prognostic analysis could not be performed. So, we only use proteomic data to further screen and validate the key genes.

In conclusion, the biomarkers we identified in multi-omics analysis were associated with sensitivity of nCT and prognosis. They require further prospective investigation and are expected to promote nCT standardization and personalization.

## Data availability statement

Publicly available datasets were analyzed in this study. These datasets can be found here: TCGA database (http://cancergenome.nih.gov/), DISNOR (https://disnor.uniroma2.it/) (32), The CPTAC database (https://proteomics.cancer.gov/programs/cptac), The UALCAN web resource (http://ualcan.path.uab.edu/). The sequencing data were deposited in the The Genome Sequence Archive for Human, accession number HRA002714 (http://bigd.big.ac.cn/gsa-human).

## Ethics statement

The studies involving human participants were reviewed and approved by The Ethical Committees of Harbin Medical University Cancer Hospital. The patients/participants provided their written informed consent to participate in this study.

## Author contributions

B-BC and Y-LL designed the study. X-FJ, B-MZ, F-QD and J-NG drafted the manuscript, collected, analyzed, and interpreted the data. DW, Y-EL and S-HD drew the figures. B-BC and Y-LL helped with the final revision of the article. All authors contributed to the article and approved the submitted version.

## Supplementary material

Supplementary Figure 1 Correlation analysis in the expression of therapeutic relevant genes with sensitivity of chemotherapy drugs. **(A)** The increased expression of genes with CNV relatively gained in the poor group was associated with decreased sensitivity to multiple chemotherapeutic drugs, including AURKA and BRAF. **(B)** In the better group, increased expression of genes with CNV relatively gained was associated with increased sensitivity to multiple chemotherapeutic drugs, including EGFR, SRC, and HSP90AA1.

Supplementary Figure 2 The changes of RNA expression of the 8 genes identified from the Venn diagram at different CNV levels, including HSP90AA1 **(A)**, MDM4 **(B)**, MTOR **(C)**, SRC **(D)**, ARID1A **(E)**, AURKA **(F)**, BRAF **(G)** and EGFR **(H)**.

Supplementary Figure 3 Immunohistochemical staining for the key genes EGFR and HSP90AA1in normal tissues, READ tissues and COAD tissues. (Image credit: Human Protein Atlas, images available from v20.1.proteinatlas.org).
