# Supplementary figures and images for: Exploring biomarkers for prognosis and neoadjuvant chemosensitivity in rectal cancer: Multi-omics and ctDNA sequencing collaboration
Source: Front Immunol. 2022 Dec 9;13:1013828. doi: 10.3389/fimmu.2022.1013828 (PMC9780298; doi:10.3389/fimmu.2022.1013828)

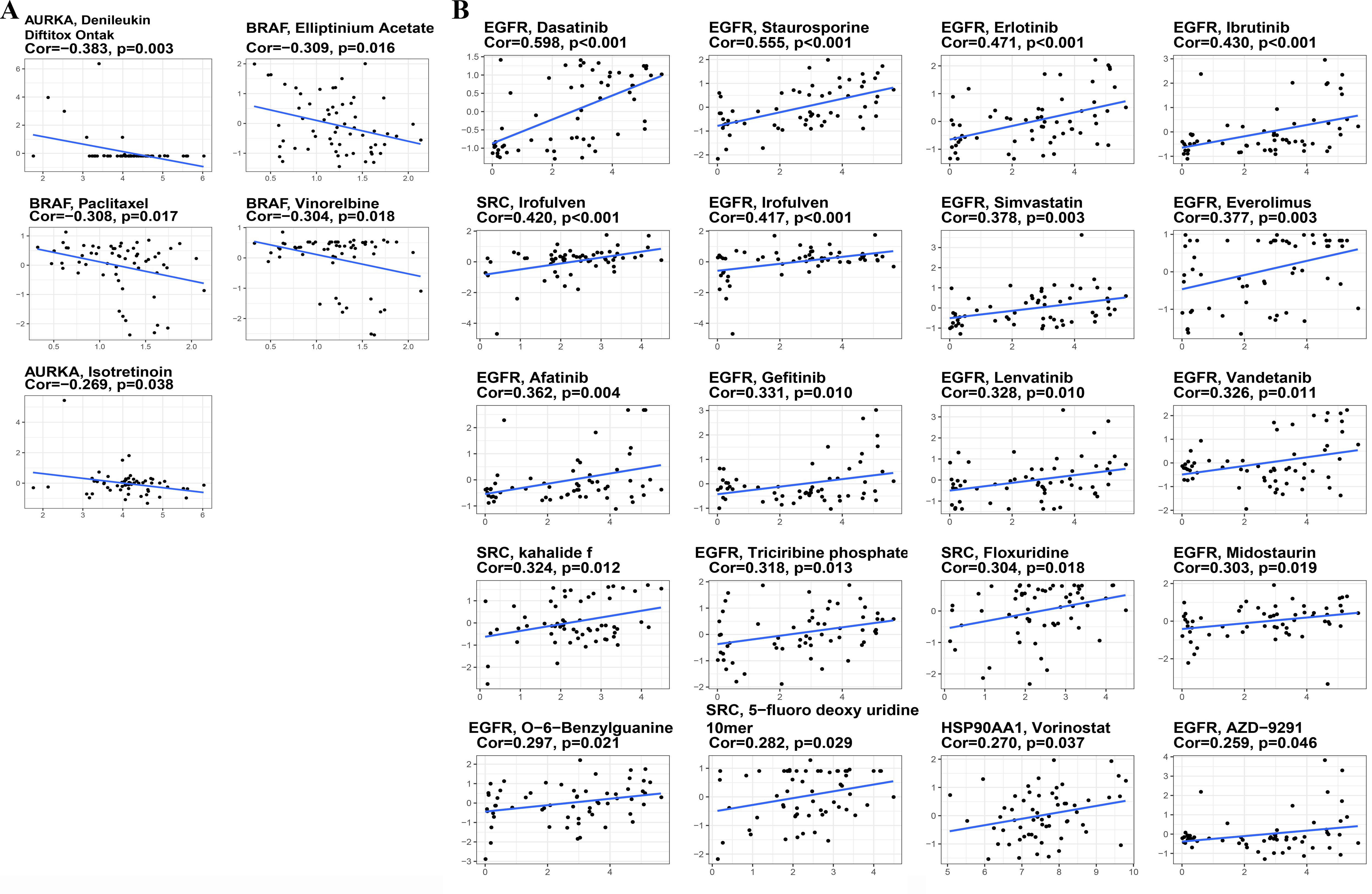

Supplement: Supplementary Figure 1 — Correlation analysis in the expression of therapeutic relevant genes with sensitivity of chemotherapy drugs. (A) The increased expression of genes with CNV relatively gained in the poor group was associated with decreased sensitivity to multiple chemotherapeutic drugs, including AURKA and BRAF. (B) In the better group, increased expression of genes with CNV relatively gained was associated with increased sensitivity to multiple chemotherapeutic drugs, including EGFR, SRC, and HSP90AA1. [file Image_1.tiff]

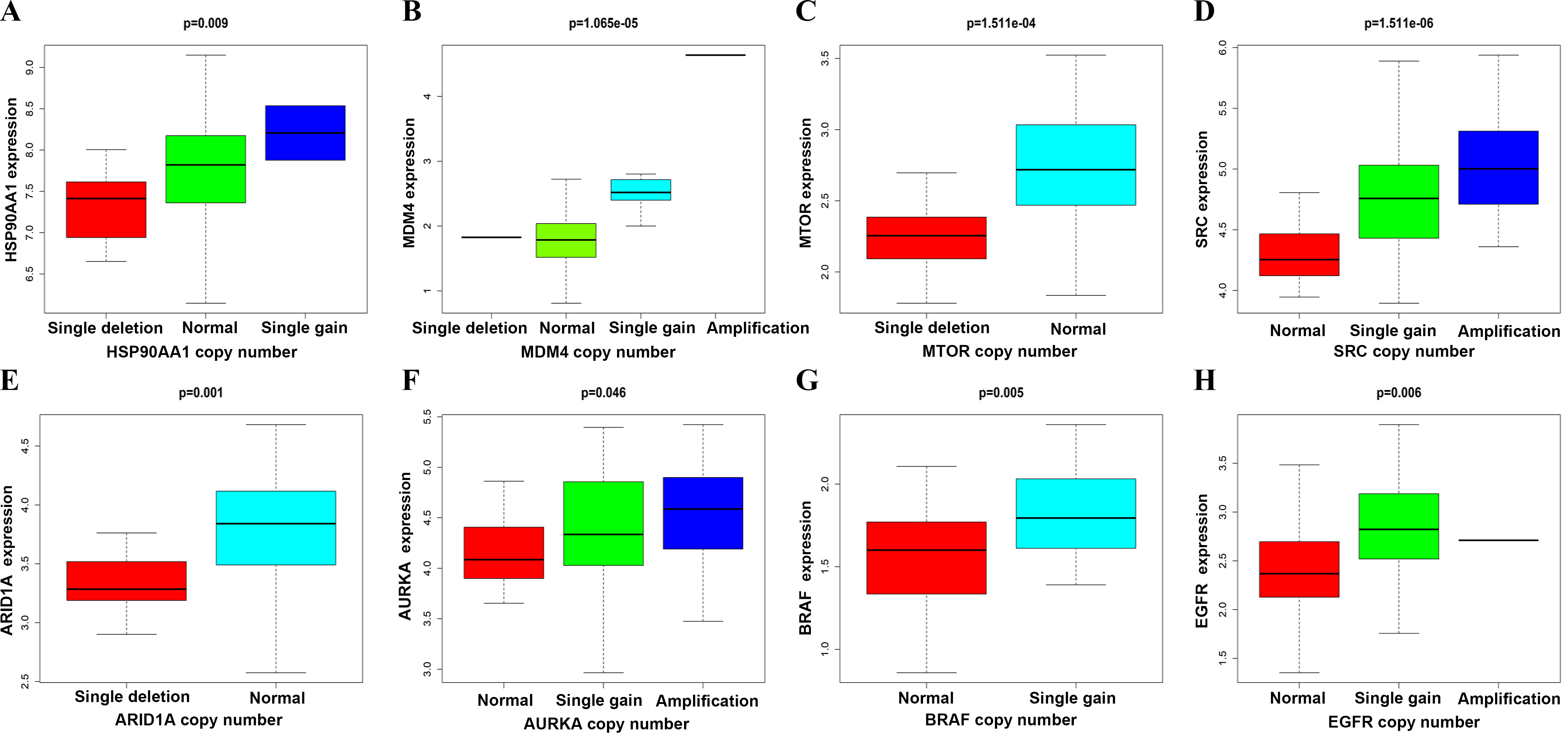

Supplement: Supplementary Figure 2 — The changes of RNA expression of the 8 genes identified from the Venn diagram at different CNV levels, including HSP90AA1 (A), MDM4 (B), MTOR (C), SRC (D), ARID1A (E), AURKA (F), BRAF (G) and EGFR (H). [file Image_2.tiff]

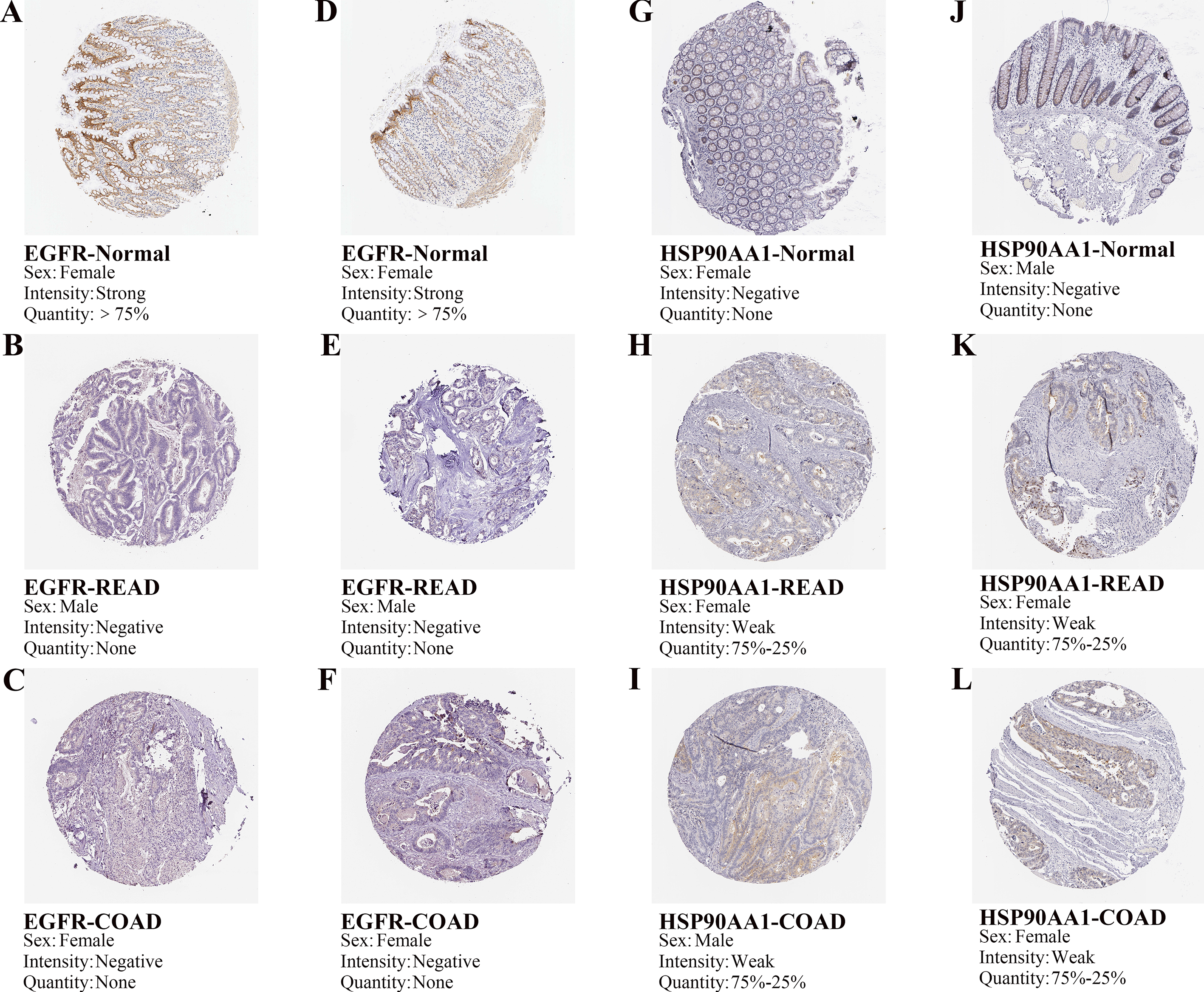

Supplement: Supplementary Figure 3 — Immunohistochemical staining for the key genes EGFR and HSP90AA1in normal tissues, READ tissues and COAD tissues. (Image credit: Human Protein Atlas, images available from v20.1.proteinatlas.org). [file Image_3.tif]
